# Typhoid Fever of Children; Ibid

**Published:** 1878-08-20

**Authors:** 


### TYPHOID FEVER OF CHILDREN.

In the typhoid fever of children he gives, to a child six years of age, a teaspoonful every three hours of a three per cent. solution of the salicylate of soda.

If the temperature does not run higher than 38.3° C., he uses no other treatment except nourishment in a fluid form, and red claret wine in doses according to the tendency that exists to a failure of the heart's action. Whenever the thermometer in the axilla registers a temperature above 38.3° C., he then resorts to the use of cold packing with water ranging from 13° C. to 18° C. If the case be a more severe one, and the temperature runs as high as 40° C. to 40.5° C., he resorts to cold baths, putting the patient into water at 30° C., and then adding cold water to reduce it from 18° to 24° C. These baths are only used as long as the fever is very high, the patient being kept in them from 10 to 30 minutes, according to the time necessary to reduce the temperature.

Speaking of the effects of quinia in fevers (upon which subject the doctor remarked that the average physician was was a maniac), he only gives the remedy in their later stages, and then in small doses as a tonic.

To illustrate in what manner quinia acts as an anti-pyretic, he related the following case:

"I was called a few days ago in consultation with another physician to see a child aged ten years, suffering from typhus abdominalis. As the attending physician was fond of giving quinia in this disease, I finally consented that the patient might have 6 decigrams of the drug, divided into three doses, and given at intervals of three hours, just before the exacerbation of the fever. The physician not understanding the metric system of weights (it having been introduced into Austria only a few years), wrote his prescription for 6 *grams* instead of *decigrams*." Dr. Monti was called again soon after the child had taken the last dose, and found it was deaf as an adder, breathing heavily, and capable of being aroused only to semi-consciousness with great difficulty. The temperature was reduced to 35.5 deg. C. in the axilla, though before the remedy was taken it was 40 deg. C. Stimulants were given, cold was applied to the head, and warm applications to the feet, and after some hours the patient recovered from the effects of the drug. In this case, although there was such a marked reduction of temperature at the time, the following day the exacerbation of the fever was the same as before the remedy was given.

The inference drawn by Dr Monti from this case, as well as from an extensive trial of the drug in other cases of typhoid fever, is that quinia only acts as a *decided* anti-pyretic when given in doses that must be considered as *poisonous* in size, and that even when these doses are given daily, they exercise no influence in shortening the duration of the attack.

As bearing on this question of the anti-pyretic effects of quinia in this disease, I will give the following case from my own private note-book. During the fall of 1875, while we were having a severe epidemic of typhoid fever in Warren, Illinois, I was called to attend the daughter, aged 13 years, of Mr. H——, a lawyer of that town.

The patient had enjoyed previous good health, and was large and well developed for her age.

The attack was severe from the outset, the temperature running to 40 deg. C. after the first week, and rising to 40.5 deg. C. after the middle of second, and was accompanied by marked cerebral disturbance. My plan has always been to combat vigorously a temperature that reaches or exceeds 40 deg. C. For this purpose I usually wring cloths from water varying from 15 deg. to 20 deg. C., and envelop the body from under the axilla to the knees.

If the patient be an adult, the cloth used should be as large as a common sheet.

I direct that these cloths should be changed as often as every 15 to 20 minutes, until the temperature is brought down to a point that I consider compatible with the safety of the patient, say to 38 deg. or 39 deg. C.

To use this remedy requires considerable judgment on the part of the nurse, with whom I always leave a thermometer, so that my cold applications may be used intelligently.

As sometimes happens, the case above referred to was very sensitive to the application of the wet pack, and so after trying it in a rather unsatisfactory manner for a week, I determined to discontinue its use, and resort to quinia to fulfill the indications for which I had been using the cold water.

With this view I gave her 0.48 gram of the remedy at 4, 6 and 8 o'clock p. m., for three days in succession. The effect of the drug was to increase the nervous disturbance with my patient, without in the least lessening the temperaeure of the body, and finding her in every way worse at the expiration of this time, I fell back with renewed energy on my hydropathic treatment of the case.

From this time on I gave no medicine save a single dose of chloral-hydrate at night, kept the temperature down to 38.3 deg. C., and my patient grew gradually better and made a good recovery, after a duration of the fever of eighteen days.

In Prof. Duchek's wards here in Vienna, to which, by the courtesy of his first assistant, Dr. Brenner, I have had free access at all times, the treatment of the milder cases of typhoid fever is entirely expectant.

The patient is given small doses of dilute sulphuric acid, but as this is their universally prescribed *placebo*, it must not be considered in the light of an attempt at medication.

One point in their dietetic rules here is worthy of note, and that is that no patient who has typhoid fever of even the mildest type, is allowed a single mouthful of food as long as he has any increase of temperature. Milk, soup and claret wine are used exclusively as nourishment.

When the case is more severe, and any treatment is considered necessary, either salicylate of soda or quinia is given.

The former is preferred in the earlier stages of the disease, or where there exists any marked tendency to cerebral hyperæmia.

The manner of prescribing the remedy is as follows:

| | |
|---|---|
| Salicylate of soda.................. | 5 |
| Syrup of raspberry................. | 20 |
| Water.................................. | 200 Mix. |

Give one tablespoonful every 3 hours during the waking hours of the patient, or from 14 to 16 hours out of the twenty-four.

When quinia is used, 1 gram is given in the twenty-four hours, usually in three doses, at 2, 5 and 8 p. m.

Cathartics are not given, the bowels being moved every three or four days by injections.

But it is to the manner of using cold water in these wards that I wish to call the attention of the reader, as it is entirely practicable, and its value such as after considerable experience I have verified in my own practice.

First, baths are entirely ignored.

The arguments against them, are:—first, that their use involves an amount of discomfort to the patient, that is not compensated for by any additional advantage that they offer over the wet sheet.

Second, in using them it is necessary to subject the patient to an amount of physical exertion that is likely to act deleteriously on the future course of the disease. In other words, they deem it essential that the patient should be kept as absolutely quiet as possible during the whole course of the fever.

If he be feeble, in the latter stages of the disease, he is not allowed to rise upright in bed, even to evacuate the bladder or bowels.

This perfect quiet is supposed to retard the fatty metamorphosis in the muscular tissue of the heart and other organs.

Fever patients here are usually dressed in a single garment, open in front, reaching to the feet, somewhat after the fashion of a lady's night-dress. A rubber sheet is put over the mattress to prevent its becoming damp. When the temperature rises to a point that is thought to be unsafe, they resort to the cold pack, and use it in the following manner. The patient, being dressed as above indicated, a sheet is wrung out of water of a temperature varying from 7.5 deg. to 15.5 C., and in this his entire body is enveloped, and over this is thrown a dry covering. These sheets are changed every 14 to 20 minutes for from two to five times, a thermometer being kept constantly in the axilla to denote the fall in temperature that is required.

After the temperature has fallen, the patient's garment as well as his bedding is changed, for the sake of dryness.

By the assistance of two intelligent nurses, all this is accomplished with the least possible amount of exertion on the part of the patient.

If he be decidedly feeble, he is given either brandy alone or milk punch before the wet sheets are used.

The advantage of the procedure above given is that it can be carried out easily in private practice, which is not the case with the cold baths as recommended by Leibermeister.—*Ib.*

